# Corrigendum

**DOI:** 10.1002/ueg2.12258

**Published:** 2022-05-31

In the article entitled “Guideline for the diagnosis and treatment of Faecal Incontinence—A UEG/ESCP/ESNM/ESPCG collaboration”,^1^ in Figure 1, the box ‘second line: surgical interventions’ should only include 3 treatment options, rather than 4 and it has been revised as below:

**FIGURE 1:**
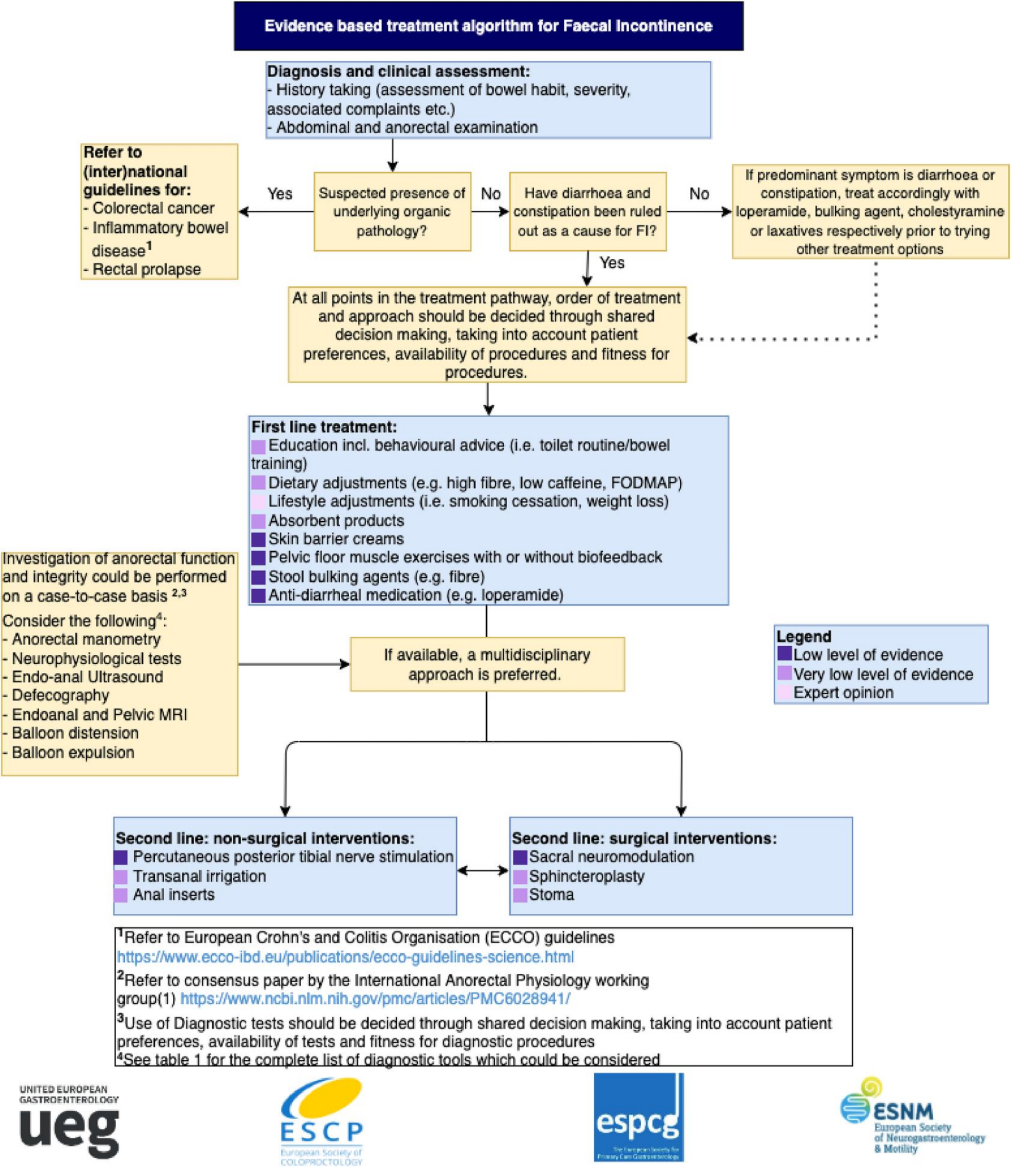
Treatment algorithm Faecal Incontinence

We apologize for this error.

## References

[1] Assmann SL , Keszthelyi D , Kleijnen J , Anastasiou F , Bradshaw E , Brannigan AE , et al. Guideline for the diagnosis and treatment of Faecal Incontinence‐A UEG/ESCP/ESNM/ESPCG collaboration. UEG Journal. 2022;10(3):251–86. 10.1002/ueg2.12213 35303758 PMC9004250

